# Mining, Validation, and Clinical Significance of Colorectal Cancer (CRC)-Associated lncRNAs

**DOI:** 10.1371/journal.pone.0164590

**Published:** 2016-10-27

**Authors:** Xiangwei Sun, Yingying Hu, Liang Zhang, Changyuan Hu, Gangqiang Guo, Chenchen Mao, Jianfeng Xu, Sisi Ye, Guanli Huang, Xiangyang Xue, Aizhen Guo, Xian Shen

**Affiliations:** 1 Department of General Surgery, First Affiliated Hospital, Wenzhou Medical University, Wenzhou, China; 2 Department of Obstetrics and Gynecology, Second Affiliated Hospital, Wenzhou Medical University, Wenzhou, China; 3 Department of Microbiology and Immunology, Institute of Molecular Virology and Immunology, Institute of Tropical Medicine, Wenzhou Medical University, Wenzhou, China; 4 Department of Surgical Oncology, First Affiliated Hospital, Wenzhou Medical University, Wenzhou, China; 5 Department of Internal Medicine, Yangpu Hosptial, Tongji University School of Medicine, Shanghai, China; 6 Department of General Surgery, Second Affiliated Hospital, Wenzhou Medical University, Wenzhou, China; Washington State University, UNITED STATES

## Abstract

**Background:**

Colorectal cancer (CRC) is one of the deadliest tumours, but its pathogenesis remains unclear. The involvement of differentially expressed long non-coding RNAs (lncRNAs) in CRC tumorigenesis makes them suitable tumour biomarkers.

**Methods/Findings:**

Here, we screened 150 cases of CRC and 85 cases of paracancerous tissues in the GEO database for differentially expressed lncRNAs. The levels of lncRNA candidates in 84 CRC and paracancerous tissue samples were validated by qRT-PCR and their clinical significance was analyzed. We identified 15 lncRNAs with differential expression in CRC tumours; among them, AK098081 was significantly up-regulated, whereas AK025209, BC040303, BC037331, AK026659, and CR749831 were down-regulated in CRC. In a receiver operating characteristic curve analysis, the area under the curve for the six lncRNAs was 0.914. High expression of AK098081 and low expression of BC040303, CR749831, and BC037331 indicated poor CRC differentiation. CRC patients with lymph node metastasis had lower expression of BC037331. In addition, the group with high AK098081 expression presented significantly lower overall survival and disease-free survival rates than the low-expression group, confirming AK098081 as an independent risk factor for CRC patients.

**Conclusion/Significance:**

In conclusion, we have identified multiple CRC-associated lncRNAs from microarray expression profiles that can serve as novel biomarkers for the diagnosis and prognosis of CRC.

## Introduction

Colorectal cancer (CRC) is the third most common malignant cancer, causing more than 693,900 deaths worldwide each year [1]. In China, CRC is the fourth most common malignant tumour, with a progressively increasing incidence [2]. With recent advances of comprehensive CRC treatment, more than 90% of patients with early-stage CRC can now be cured. However, in most CRC patients, the tumour has already developed to an advanced stage by the time it is diagnosed, thus severely reducing the five-year survival rate [3]. The identification of new molecular regulatory mechanisms in CRC tumorigenesis and progression could result in novel therapeutic targets for the diagnosis and treatment of CRC.

In the post-genomic era, transcriptomics studies have gradually gained importance. More than 98% of genome transcripts are non-coding RNAs (ncRNAs) that do not encode for proteins. Among them, long non-coding RNAs (lncRNAs) are ncRNAs of more than 200 nucleotides. LncRNAs can regulate physiological and pathological processes at epigenetic, transcriptional, and post-transcriptional levels [4]. Numerous studies have shown that lncRNAs are involved in cell proliferation, differentiation, apoptosis, metastasis, and many other processes. Given their significant role in tumorigenesis and development, they have become of interest in cancer research. Liu et al.reported that over-expression of NF-κB-interacting lncRNA-NKILA suppressed breast cancer metastasis [5]. Zhang et al. found that over-expression of lncRNA-TUG1 promoted the growth of gastric cancer cells and was associated with poor prognosis of this disease [6]. In addition, lncRNA-FEZF1-AS1 has been found to mediate CRC tumorigenesis and progression [7], whereas lncRNA-CASC11 appears to be up-regulated in CRC, where it promotes proliferation and metastasis [8]. Considering that lncRNAs closely correlate with the clinical characteristics of cancer patients, they can be used as diagnostic and prognostic tumour indicators [9, 10].

The screening of relevant lncRNAs in tissue samples is mainly based on lncRNA microarrays [11]. To date, the amount of open-access lncRNA microarrays for CRC samples is limited. Over the past 20 years, differentially expressed genes in CRC have been detected using microarray expression profiles. These data are now deposited in public databases such as the Gene Expression Omnibus (GEO). Recent studies have shown that these data sets encompass a large number of specific probes, which are now considered as lncRNAs [12]. In this study, we systematically searched the GEO database for differentially expressed lncRNAs in CRC and paracancerous tissues. The levels of lncRNAs in CRC and paracancerous tissues were validated by quantitative real-time polymerase chain reaction (qRT-PCR) and correlated with the clinicopathological features of CRC.

## Materials and Methods

### Mining of lncRNA data from microarrays

We first searched the GEO database (http://www.ncbi.nlm.nih.gov/gds/) for all HGU133PLUS2.0 microarray data related to CRC. Data were revised to exclude instances of less than 10 cancerous and paracancerous samples per group. IQRray_score [13] and presence/absence call [14] were used to ensure the overall quality of microarray data and exclude any abnormal specimens. A platform called GATExplorer [15] was used to analyze microarray data at the nucleotide level in a genomic context and distinguish lncRNA candidates.The standards for lncRNAs in GATExplorer were:(i) there was experimental (i.e. EST, cDNA, RT–PCR and/or northern blot) evidence to support their existence as RNAs; (ii) they did not contain a significant ORF (i.e. <100 amino acids); (iii) they were not annotated as rRNAs, tRNAs, snoRNA, miRNA and spliceosomal RNAs; and (iv) they were mammalian [16]. Mircoarray-meta analysis [17] was applied to identify differentially expressed lncRNAs in the cancerous and paracancerous tissues.

### Tissue specimens

Pathologically confirmed tissue specimens from 84 patients who had not received radiation or chemotherapy before surgery were collected at The First Affiliated Hospital of Wenzhou Medical University between February 2004 and December 2008. The specimens were obtained from surgically removed cancerous and corresponding paracancerous normal tissues, and stored in liquid nitrogen with long-term follow-up records. Paracancerous normal tissues means the normal mucosa tissue witch is more than 5cm away from the edge of cancer. The study was approved by the Research Ethics Committee of The First Affiliated Hospital of Wenzhou Medical University. Written informed consent was obtained from all patients. Patient information can be found in S1 Table. The overall survival time was calculated from the date of surgery to death. Disease-free survival time was calculated from the date of diagnosis to the date of surgery and recurrence; if recurrence was not diagnosed, then the date of death or the last follow-up was used as a reference.

### 5'/3'rapid amplification of cDNA ends (RACE)

Total RNA was extracted from frozen specimens using TRIzol (Life Technologies, Carlsbad, CA, USA) according to the manufacturer’s instructions. RNA concentration was determined using a UV-visible spectrophotometer (NanoDrop ND-1000). Oligo dT-3' Adaptor primers from the 3'-Full Race Core set (TaKaRa, Shiga, Japan) were used for reverse transcription of total RNA into cDNA. To obtain the lncRNA 3'-end fragment, nested PCR was performed using a set of nested primers, consisting of a specific primer pair (S2 Table) and the 3'RACE-Out and 3'RACE-Inner primers from the kit. Total RNA was dephosphorylated and “decapitated” as instructed in the 5'-Full RACE Kit (TaKaRa), followed by ligation of the 5' Adaptor primer and reverse transcription. To obtain the lncRNA 5'-end fragment, a nested PCR was performed using a set of nested primers, consisting of a specific primer pair (S2 Table) and 5'RACE-Out and 5'RACE-Inner primers from the kit. All fragments obtained were sent to the Beijing Genomics Institute (Beijing, China) for sequencing after ligation in the pMD18-T vector (TaKaRa).

### Quantitative real-time PCR assay

Total RNA (1μg) was used as template for reverse transcription using the ReverTraAce qPCR RT kit (Toyobo, Tokyo, Japan). LncRNA was quantified by RT-PCR using RNA-direct SYBR Green Real-time PCR Master Mix (Toyobo) and specific primers. Reactions were performed on a CFX96 Touch™ Real-Time PCR Detection System (Bio-Rad, Hercules, CA, USA) under the following conditions: initial step of 5 min at 95°C, followed by 40 amplification cycles of 15 s at 95°C, annealing for 32 s at a custom temperature, and a final step of 32 s at 72°C. Primers were obtained from the Beijing Genomics Institute. Primer sequences and annealing temperatures can be found in S2 Table. Each experiment was performed in triplicate.

### Statistical Analysis

All data were processed and statistically analyzed using SPSS software version 21 (SPSS, Chicago, IL, USA). The differential expression of lncRNAs in CRC and paracancerous normal tissues was examined using the paired *t*-test.The correlation between lncRNA expression and clinicopathological features was analyzed using the unpaired *t*-test or analysis of variance (≥ 3 groups). Univariate analysis and multivariate forward logistic regression analysis were used to construct the logistic regression model of lncRNA for prediction of CRC. Survival curves were analyzed using the Kaplan-Meier method and statistical significance was determined using the log-rank test. The significance of survival variables was analyzed by the Cox multivariate proportional hazards model. P<0.05 was considered statistically significant.

## Results

### Mining of lncRNA data from microarrays for expression profiling of CRC

**Fig 1:**
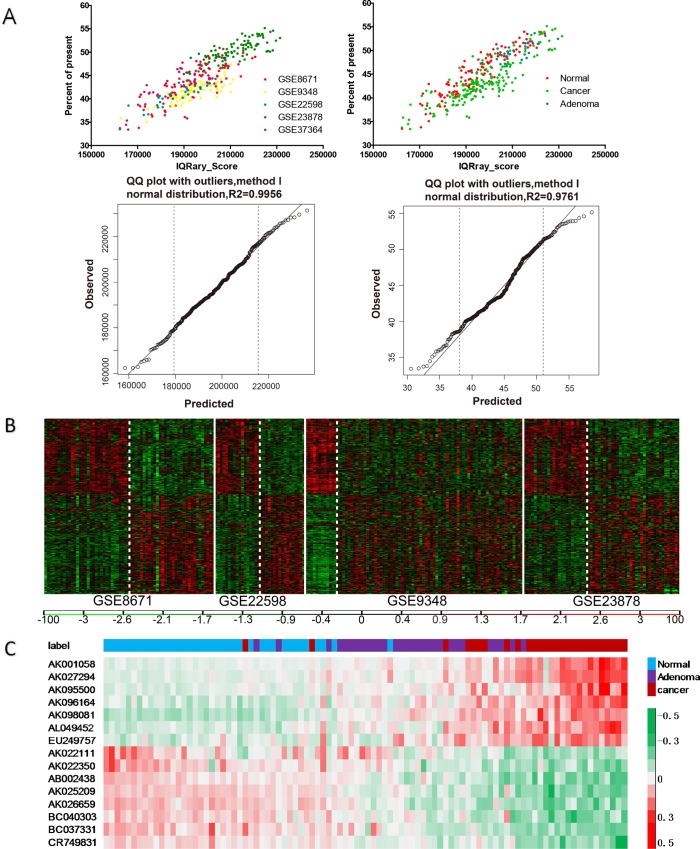
Screening of differentially expressed lncRNAs. (A) IQRray_score and presence/absence call were used for microarray quality assessment to ensure the overall quality of microarray data without any abnormal specimens. (B) 142 differentially expressed lncRNAs were selected from GSE8671, GSE22598, GSE23878, and GSE9348, P≤0.01. (C) The 142 lncRNAs were then compared to GSE37364, which included 27 cases of CRC, 29 cases of colorectal adenoma, and 38 cases of normal tissues. As a result, 15 lncRNAs differentially expressed in cancerous, adenoma, and normal tissues were selected, FDR<0.01. Red indicateshigh relative expression, and green indicates low relative expression.

In total of 1725 HGU133PLUS2.0 microarray data sets involving CRC were found in the GEO database. Among them, five studies were selected: GSE8671, GSE22598, GSE23878, GSE9348, and GSE37364 (Table 1). Following microarray quality control (Fig 1A), 142 differentially expressed lncRNAs with P≤0.01 were selected from GSE8671, GSE22598, GSE23878, and GSE9348 [18]. These lncRNAs included 150 cases of CRC and 85 cases of paracancerous tissue specimens (Fig 1B),and they were then compared to GSE37364, which included 27 cases of CRC, 29 cases of colorectal adenoma, and 38 cases of normal tissues. As a result, 15 lncRNAs differentially expressed in cancerous, adenoma, and normal tissues were selected. Among them, AK001058, AK027294, AK095500, AK096164, AK098081, AL049452, and EU249757 were gradually up-regulated whereas AK022111, AK022350, AB002438, AK025209, AK026659, BC040303, BC037331, and CR749831 were gradually down-regulated in normal colorectal, adenoma, and cancerous tissues (Fig 1C).

**Table 1:** Screening of the GEO database led to selection of five gene expression microarrays for colorectal cancer (requirements: cancer tissues and their adjacent normal tissues, at least 10 samples per group).

	Normal	Adenoma	Carcinoma	Note
GSE8671	32	/	32	paired
GSE22598	17	/	17	paired
GSE23878	24	/	35	unpaired
GSE9348	12	/	70	unpaired
GSE37364	38	29	27	unpaired

The sequences of the above-mentioned 15 lncRNA candidates were obtained from the National Center for Biotechnology Information (NCBI) database (http://www.ncbi.nlm.nih.gov/). Sequence alignment using the NONCODE (http://www.bioinfo.org/noncode/) [19] and LNCipedia (http://www.lncipedia.org) [20] databases revealed that AK001058, AK095500, AK096164, AL049452, AK025209, AK026659, BC040303, BC037331, and CR749831 fully matched existing lncRNAs, whereas EU249757 displayed only a partial match. AK098081 partly matched existing lncRNAs only in the NONCODE database (S3 Table). To determine the full length of the cellular transcriptome, we also performed 5'/3' RACE for AK096164. The 5'-end of its transcript was located at position 39 of the NCBI reference sequence, whereas its 3'-end was consistent with the reference sequence (S1 Fig). Only two known transcripts of LNC-EIF2C2-1 exist in the LNCipedia database: LNC-EIF2C2-1:1 located on chr8:14530255–141539600 and LNC-EIF2C2-1:2 located on chr8:141532118–141535841 (S2 Fig). RACE results showed that the AK096164 transcript was located on chr8:14530294–141532539, thus corresponding to a new transcript of LNC-EIF2C2-1. In summary, 11 gene candidates (AK001058, AK095500, AK096164, AL049452, AK025209, AK026659, BC040303, BC037331, CR749831, AK098081, and EU249757) were identified as lncRNAs.

### Expression of the lncRNA candidates in tissue specimens of CRC

**Fig 2:**
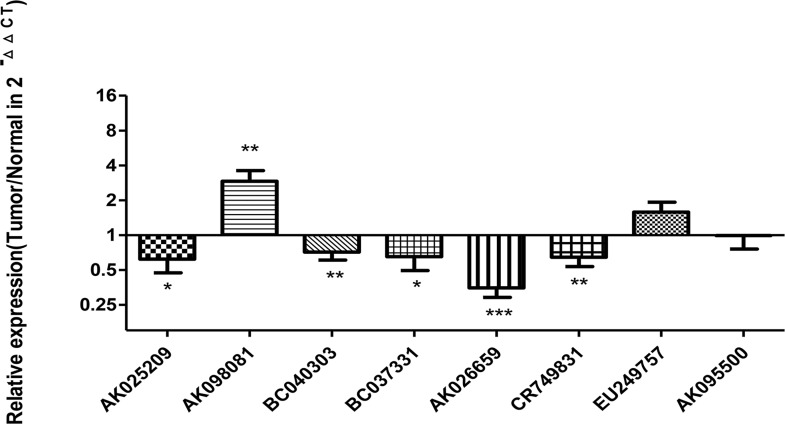
Relative expression of lncRNAs in human CRC tissues. lncRNAs levels were measured by qRT-PCR in 84 CRC tissues and corresponding paracancerous tissue. The relative expression of lncRNAs was normalized to that of GAPDH (2^-△△Ct^). The P value was obtained by using a paired Student’s t-test.(*:P<0.05;**:p<0.01; ***:p<0.001)The Ct values for AK096164, AK001058, and AL049452 were too high for statistical analysis.

The 11 lncRNAs selected by the above-mentioned experiments were further analyzed by qRT-PCR in 84 CRC and corresponding paracancerous tissue samples. The expression of lncRNAs was normalized to that of glyceraldehyde 3-phosphate dehydrogenase (GAPDH;2^-△△Ct^). The melting curve and agarose gel electrophoresis of GAPDH and each lncRNA were presented in S3 Fig. AK098081 was significantly up-regulated in CRC compared with paracancerous tissues (P<0.05); AK025209, BC040303, BC037331, AK026659, and CR749831 were significantly down-regulated in CRC (P<0.05); whereas EU294757 and AK095500 showed no significant difference in cancerous and paracancerous normal tissues (P = 0.095 and P = 0.966, respectively) (Fig 2).

### Diagnostic value of six lncRNAs as biomarkers of colorectal cancer

**Fig 3:**
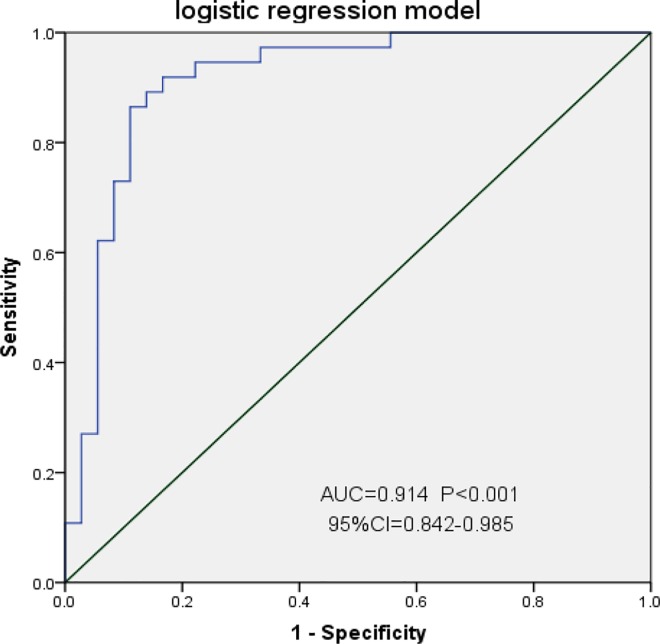
Receiver operating characteristic (ROC) curves for determing the diagnostic value of Logistic regression model. AUC, area under the ROC curve; CI, area under the ROC curve.

To determine whether AK098081, AK025209, BC040303, BC037331, AK026659, and CR749831 can be used as biological indicators for diagnosis of CRC, receiver operating characteristic (ROC) curve analysis was performed based on the expression level of lncRNAs in CRC and corresponding paracancerous normal tissues (represented as 2^-△Ct^). As shown in Fig 3, the area under the curve (AUC) values for AK098081, AK025209, BC040303, BC037331, AK026659, and CR749831 were 0.859, 0.748, 0.652, 0.667, 0.732, 0.859, and 0.738, respectively (P <0.05 for all ROC curves). Among them, AK026659 had the highest AUC diagnostic value (95% CI = 0.773–0.940), a cutoff value of 0.0419, 75.6% sensitivity, 85% specificity, and Yonden index of 0.606 (S4 Fig). To better illustrate the diagnostic value of lncRNAs for CRC, a logistic regression model was constructed. Univariate analysis of each lncRNA revealed significant results for AK026659 (P<0.001), BC037331 (P<0.008), BC040303 (P<0.015), and AK098081 (P = 0.033) (Table 2). LncRNA expression data were used to construct a multivariate forward logistic regression model. AK098081, AK026659, and BC040303 were included in the model (Table 3), with Logit(P) = 0.069+3.061xAK098081-2.896xAK026659-2.045xBC040303. The likelihood ratio test revealed χ^2^ = 34.460 and P<0.001, confirming the statistical significance of the model. This model was then applied to the existing 84 CRC patients for a prediction test and to construct the ROC curve. The AUC of the ROC curve was 0.914 (P<0.001, 95% CI = 0.842–0.985) with 86.5% sensitivity, 88.9% specificity, and a Yonden index of 0.754, thus indicating that the model had a desirable goodness of fit (Fig 3).

**Table 2:** Univariate analysis of lncRNAs associated with colorectal cancer.

lncRNA	B	S.E.	Wald	P	OR	95% CI OR	
						Lower	Upper
BC037331	-2.235	0.841	6.928	0.008	0.107	0.021	0.556
AK026659	-2.804	0.661	17.969	<0.001	0.061	0.017	0.221
AK025209	-1.267	1.446	0.768	0.381	0.282	0.017	4.794
CR749831	-1.001	0.588	2.900	0.089	0.367	0.116	1.163
BC040303	-1.938	0.794	5.968	0.015	0.144	0.030	0.683
AK098081	2.633	1.238	4.520	0.033	13.915	1.229	157.509

S.E., standard error; CI, confidence interval.

**Table 3:** Multivariate forward logistic regression analysis of lncRNAs associated with colorectal cancer.

lncRNA	B	S.E.	Wald	P	OR	95% CI OR	
						Lower	Upper
AK098081	3.061	1.336	5.249	0.022	21.349	1.557	292.821
AK026659	-2.896	0.757	14.641	0.000	0.055	0.013	0.244
BC040303	-2.045	0.809	6.383	0.012	0.129	0.026	0.632
Constant	0.069	0.639	0.012	0.913	1.072		

S.E., standard error; CI, confidence interval.

### Correlation between lncRNA levels in cancer tissues and clinicopathological characteristics of patients with colorectal cancer

Next, we analyzed the correlation between expression of the six lncRNAs (represented as 2^-△△Ct^) and CRC patients’ sex, age, tumour size, degree of differentiation, T stage, N stage, TNM stage, and serum carcinoembryonic antigen (CEA) levels. AK098081 was more highly expressed in poorly and moderately differentiated CRCs than in well-differentiated tumours (P = 0.032). The AUC of its ROC curve was 0.721 (95% CI = 0.517–0.924, P = 0.045, S6 Fig) with a cutoff value of 1.09, 78.8% sensitivity, 77.8% specificity, and Yonden index of 0.566. The low expression of BC040303 (P = 0.040), CR749831 (P = 0.022), and BC037331 (P = 0.037) indicated that the tumour was poorly differentiated. Moreover, BC037331 was poorly expressed in CRC patients with lymph node metastasis (P = 0.016), and its expression was negatively correlated with serum CEA levels in CRC patients (P = 0.035) (Table 4). All clinicopathological data was presented in S3 Table.

**Table 4:** Association between the expression of lncRNAs and the clinicopathological features in 84 patients with colorectal cancer.

lncRNA	Differentiation types		P value
	Well (N = 22)	Moderate or others (N = 62)	
AK098081	1.50±0.41	3.58±0.55	0.032
BC040303	1.29±0.18	0.75±0.14	0.040
CR749831	1.13±0.25	0.61±0.10	0.022
BC037331	1.30±0.57	0.51±0.10	0.037
lncRNA	N stage^a^		P-value
	N0 (n = 46)	N1,N2(n = 38)	
BC037331	1.31±0.46	0.34±0.07	0.016
lncRNA	CEA^b^ (μg/ml)		P-value
	<5(n = 52)	≥5(n = 32)	
BC037331	1.10±0.31	0.41±0.43	0.035

The relative expression of lncRNA is expressed as 2^-△△Ct^ (mean ± SE).

a: According to NCCN Guidelines Version 2.2012 Gastric Cancer Staging

b: carcinoembryonic antigen

### Association between lncRNA expression and patient survival

**Fig 4:**
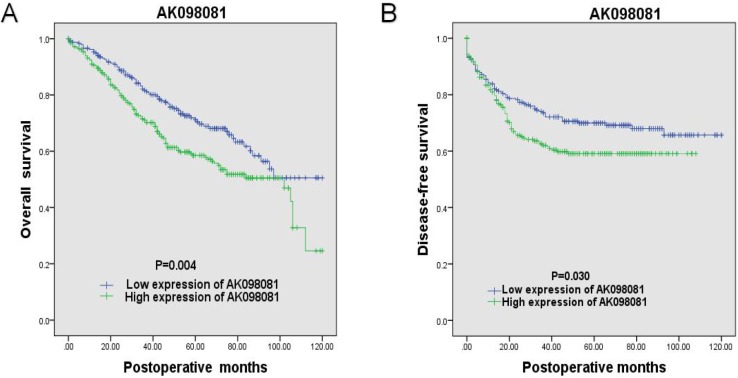
Kaplan-Meier survival curves of patients with colorectal cancer according to AK098081 expression. (A) Overall survival, (B) Disease-free survival.

The patients were divided into high- and low-expression groups based on the mean expression level of lncRNAs. LncRNAs with clinicopathological significance (AK098081, BC037331, BC040303 and CR749831) were subjected to overall survival and disease-free survival analyses. A Kaplan-Meier survival analysis showed that the group with high AK098081 expression group had significantly lower overall survival and disease-free survival rates than the low-expression group (P = 0.004 and P = 0.030, respectively, log-rank test) (Fig 4). In contrast, there was no difference in overall survival and disease-free survival rates between the high-expression and low-expression groups for BC037331, BC040303, and CR749831 (S5 Fig). To determine the prognostic capacity of AK098081 in CRC patients, we applied the Cox multivariate proportional hazards model (Table 5). Patient age, degree of differentiation of the tumour, TNM stage, serum CEA levels and AK098081 expression level were closely associated with overall survival rate (P = 0.014, P = 0.000, P = 0.001, and P = 0.000, respectively). Thus, AK098081 expression can be considered an independent risk factor for CRC patients (HR = 1.896, 95% CI = 1.393–2.579, P = 0.000).

**Table 5:** Univariate and multivariate Cox regression analysis for association of AK098081 expression with OS of patients in the study cohort (n = 84).

Factors	Overall Survival Univariate			Overall Survival Multivariate		
	HR	95% CI	P value	HR	95% CI	P value
Age(<60years, ≥60years)	1.364	0.964–1.930	0.080			
Gender(male, female)	0.738	0.550–0.989	0.042	0.682	0.504–0.925	0.014
Tumor size (<4cm, ≥4cm)	1.285	0.651–2.539	0.470			
Differentiation types(well, moderate/others)	1.944	1.332–2.838	0.001	2.022	1.394–2.931	0.000
T stage(T1/T2, T3/T4)	1.895	1.446–7.236	0.006			
N stage(N0, N1/N2)	1.511	1.123–2.033	0.070			
TNM stage(I/II, III/VI)	1.618	1.212–2.160	0.001	1.628	2.434–10.665	0.001
CEA(<5μg/ml, ≥5μg/ml)	1.517	1.337–1.876	0.045			
AK098081	1.822	1.356–2.446	0.000	1.896	1.393–2.579	0.000

HR: hazard ratio, CI: confidence interval.

## Discussion

LncRNAs are ncRNAs with a length of more than 200 nucleotides. They are involved at different levels in the regulation of physiological and pathological processes [21]. Moreover, Approximately 18% of lncRNAs are associated with tumours, whereas the proportion of tumour-associated coding genes is only 9% [22]. Hence, lncRNAs may play an important role in tumorigenesis and tumour progression [23]. Numerous studies have confirmed that lncRNAs are involved in the tumorigenesis and development of CRC via multiple pathways, such as DNA methylation and histone modifications [24, 25]. These findings have posed the question of how to accurately screen for lncRNAs of interest within the vast amount of data already available.

Compared to the traditional approach that integrated lncRNA microarrays with expression profiling, a new screening strategy enables the cost-effective integration of multiple microarrays with genome sequencing data [26]. Many of specific probes from previous expression profiling studies have been identified as putative lncRNAs [27]. Here, we searched the GEO database for all CRC expression profiling data. Five microarrays were selected, which included CRC cases, colorectal adenoma cases, and normal tissues. CRC commonly arises from normal colorectal tissue that develops into adenomas [28]. Of 142 differentially expressed lncRNAs, 15 candidates were selected.

These 15 lncRNA candidates were aligned to known lncRNAs in the NONCODE and LNCipedia databases, and 11 were identified as known lncRNAs. Interestingly, RACE experiments revealed that AK096164 lncRNA was a new transcript of LNC-EIF2C2-1. It is widely known that different lncRNA transcripts can be functionally related yet different from each other [29]. For instance, both CCAT-1 and CCAT-2 lncRNAs promote the proliferation of CRC, but CCAT-1 is an essential factor for chromosome looping at the MYC locus, whereas CCAT-2 promotes chromosomal instability [30, 31].

Quantitative RT-PCR showed that some lncRNAs (AK098081) were significantly up-regulated, whereas others (AK025209, BC040303, BC037331, AK026659, and CR749831) were down-regulated in CRC. The AUC of the ROC curves for all of these lncRNAs was 0.65–0.86, with AK026659 having the highest value, 0.859. To further improve diagnostic efficiency, the data of multiple lncRNAs were innovatively integrated to construct a logistic regression model. The AUC of the CRC-predicting model was 0.914, with 86.5% sensitivity and 88.9% specificity, indicating it had a better diagnostic efficiency than detection of serum CEA in patients [32].

Several recent studies have shown that lncRNAs are closely associated with clinicopathological features and prognosis of patients with tumours. Yunlong et al. reported that NEAT1 was highly expressed in CRC compared with paracancerous tissues. Moreover, patients with high NEAT1 expression had a poor differentiation and a shorter tumour-free survivaltime [33]. Expression of lncRNA-HOTAIR in lymph node metastatic foci of CRC was reported to be higher than that in primary tumours, indicating a poor prognosis [34]. Similarly, lncRNA-ABHD11-AS1 was found to be closely associated with tumour size and serum CEA in patients with gastric cancer [35]. In this study, the expression levels of AK098081, BC040303, CR749831, and BC037331 were associated with the degree of tumour differentiation (P = 0.032, P = 0.040, P = 0.022, and P = 0.037, respectively), whereas expression of BC037331 was negatively correlated with lymph node metastasis (P = 0.016) and serum CEA level in patients (P = 0.035). These results suggest that expression levels of AK098081, BC037331, BC040303, and CR749831 are associated with poor prognosis in CRC patients. We also found that overall survival and disease-free survival rates were lower in the group with over-expression of AK098081 than in the low-expression group, confirming that AK098081 is an independent risk factor for CRC patients. Therefore, the lncRNAs identified in this study can be used as new biomarkers to predict and even improve the prognosis of CRC patients.

In summary, we have applied an innovative data mining approach to rapidly screen for differentially expressed lncRNAs in CRC using published gene expression profiling microarrays. We have also integrated multiple lncRNA expression data to construct a predictive model for CRC. This method enabled us to analyse the clinicopathological significance of lncRNAs and their effects on the prognosis of CRC patients. The present study has successfully demonstrated that the identified lncRNAs are potential diagnostic and even therapeutic targets in CRC.

## Supporting Information

S1 Fig Cloning of the full-length human AK096164. (A) Left, representative image of nested PCR products from the 5'RACE procedure. The major PCR product is marked. Right, sequencing of the second-round PCR products revealed the boundary between the 5'RACE-Inner primer and the reverse complement sequences of AK096164. The thymine marked by an arrow indicates a putative transcriptional start site. (B) Left, representative image of nested PCR products from the 3'RACE procedure. Right, sequencing of the second-round PCR products revealed the boundary between the 5'RACE-Inner primer and the AK096164 sequences. The cytimidine marked by an arrow indicates a putative transcriptional termination site. (C): The nucleotide sequence of the full-length human AK096164 gene is shown, the arrow represents the transcriptional start site identified by 5'RACE, and the triangle represents the transcriptional termination site identified by site identified by 3'RACE.
(TIF)

S2 Fig AK096164 is a new transcripts of LNC-EIF2C2-1. The AK096164 sequence identified by RACE, which is located at chr8: 14530294–141532539, is a new transcript of LNC-EIF2C2-1, according to the LNCipedia database.
(TIF)

S3 Fig Melting curve and agarose gel electrophoresis of the GAPDH transcript and each lncRNA. (A) The melting curve of the GAPDH transcript and each lncRNA. (B) Agarose gel electrophoresis of the GAPDH transcript and each lncRNA. 1–5: BC040303, CR749831, AK026659, GAPDH, BC037331; 6–9: AK095500, EU249757, AK098081, AK025209.
(TIF)

S4 Fig Receiver operating characteristic (ROC) curve of patients with colorectal cancer based on lncRNA expression in tumour tissues and non-tumour adjacent tissues. AUC: area under the ROC curve, CI: confidence interval.
(TIF)

S5 Fig Kaplan-Meier survival curves of patients with colorectal cancer according to CR749831,AK001058 and BC037331 expression. (A) Correlation of overall survival and disease-free survival with CR749831 expression. (B) Correlation of overall survival and disease-free survival of AK001058 expression. (C) Correlation of overall survival and disease-free survival of BC037331 expression.
(TIF)

S6 Fig Receiver operating characteristic (ROC) curve of CRC differentiation status based on AK098081 expression level. (TIF)

S1 Table All clinicopathological analyses for lncRNAs and CRC. (XLSX)

S2 Table Primer for rapid amplification of cDNA ends (RACE) and quantitative real-time PCR assays. (XLSX)

S3 Table LncRNA sequences were aligned with the NONCODE database and the LNCipedia database. (XLSX)
