# Supplementary figures and images for: Mining, Validation, and Clinical Significance of Colorectal Cancer (CRC)-Associated lncRNAs
Source: PLoS One. 2016 Oct 27;11(10):e0164590. doi: 10.1371/journal.pone.0164590 (PMC5082825; doi:10.1371/journal.pone.0164590)

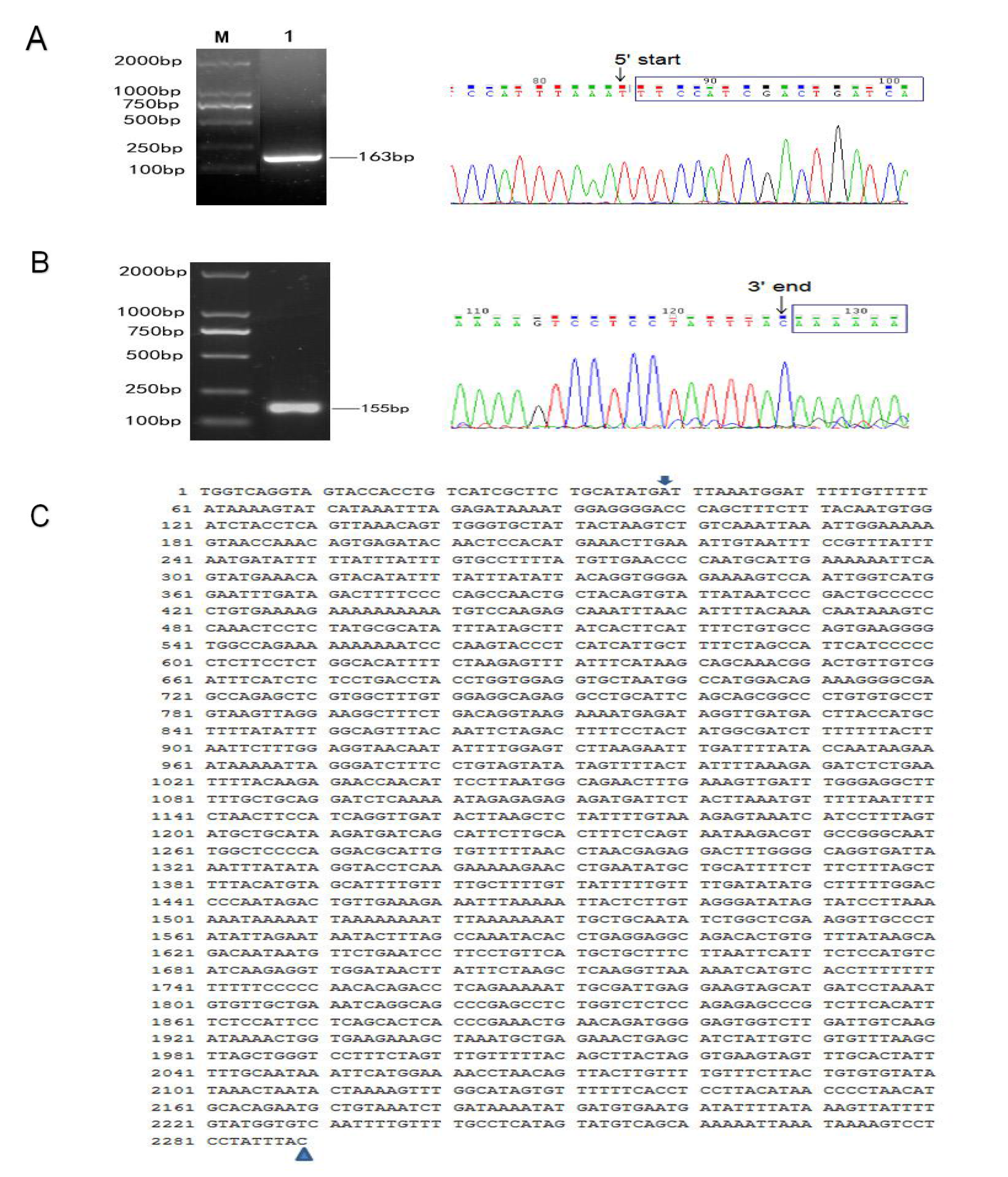

Supplement: S1 Fig — (A) Left, representative image of nested PCR products from the 5'RACE procedure. The major PCR product is marked. Right, sequencing of the second-round PCR products revealed the boundary between the 5'RACE-Inner primer and the reverse complement sequences of AK096164. The thymine marked by an arrow indicates a putative transcriptional start site. (B) Left, representative image of nested PCR products from the 3'RACE procedure. Right, sequencing of the second-round PCR products revealed the boundary between the 5'RACE-Inner primer and the AK096164 sequences. The cytimidine marked by an arrow indicates a putative transcriptional termination site. (C): The nucleotide sequence of the full-length human AK096164 gene is shown, the arrow represents the transcriptional start site identified by 5'RACE, and the triangle represents the transcriptional termination site identified by site identified by 3'RACE. (TIF) [file pone.0164590.s001.tif]

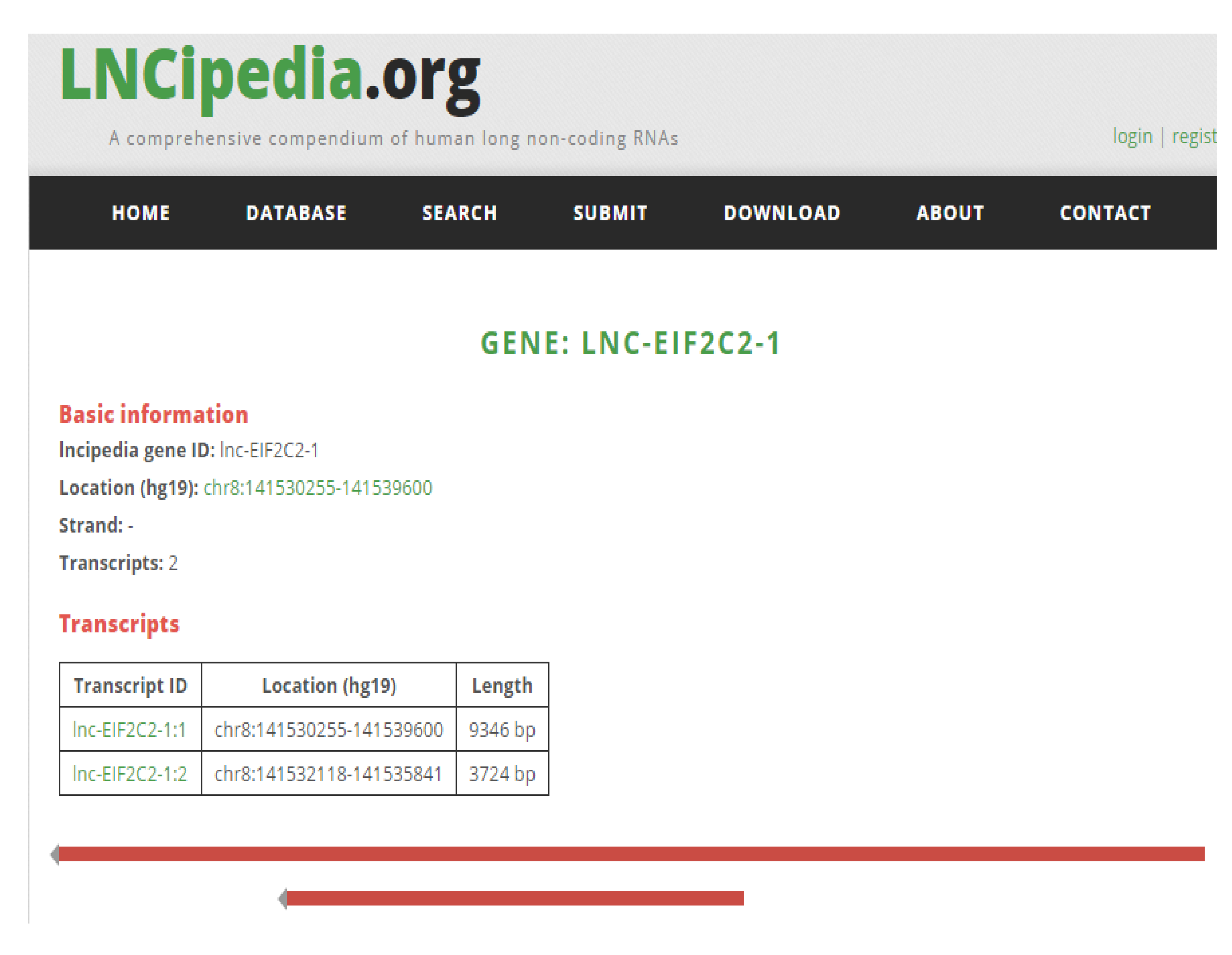

Supplement: S2 Fig — The AK096164 sequence identified by RACE, which is located at chr8: 14530294–141532539, is a new transcript of LNC-EIF2C2-1, according to the LNCipedia database. (TIF) [file pone.0164590.s002.tif]

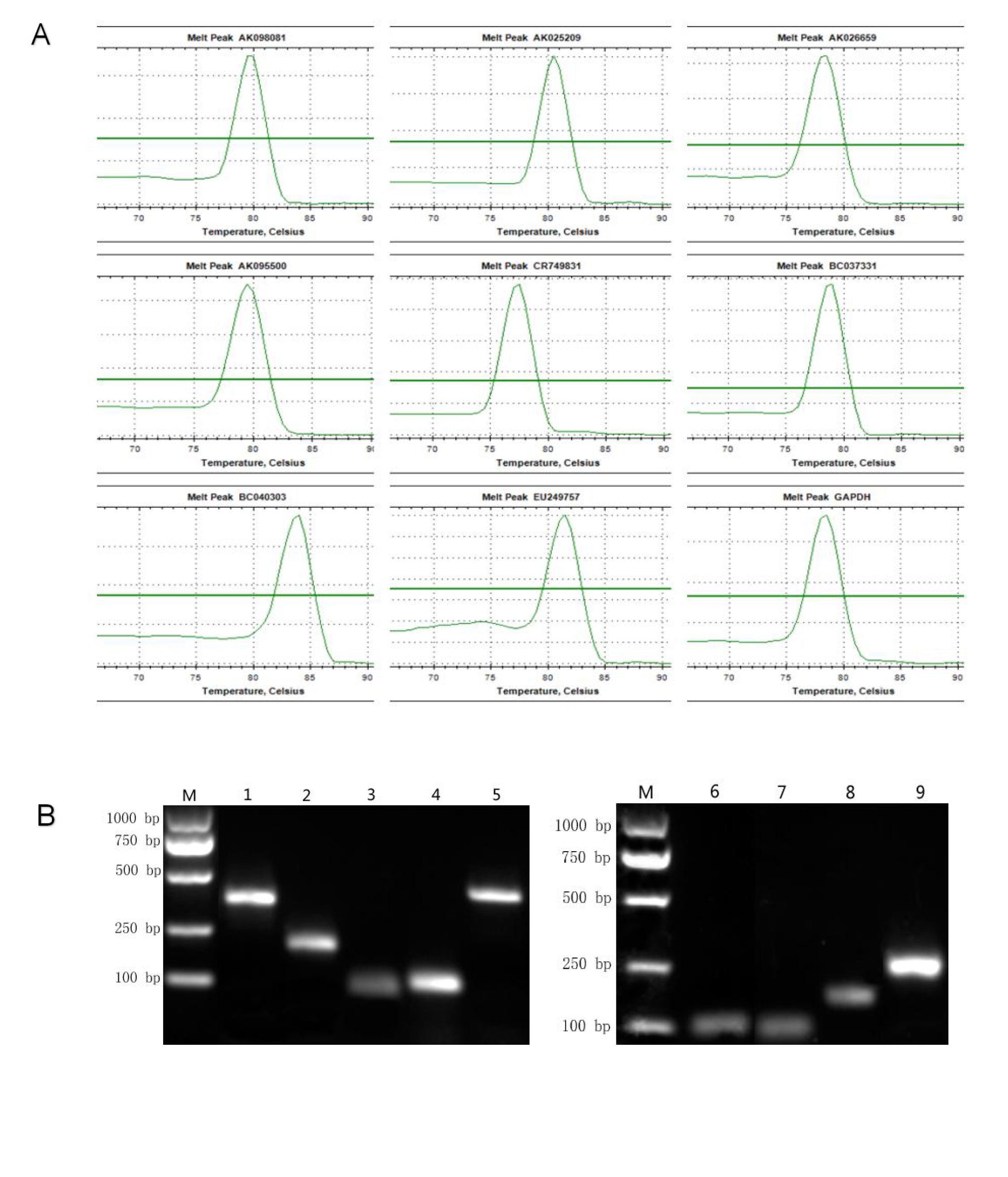

Supplement: S3 Fig — (A) The melting curve of the GAPDH transcript and each lncRNA. (B) Agarose gel electrophoresis of the GAPDH transcript and each lncRNA. 1–5: BC040303, CR749831, AK026659, GAPDH, BC037331; 6–9: AK095500, EU249757, AK098081, AK025209. (TIF) [file pone.0164590.s003.tif]

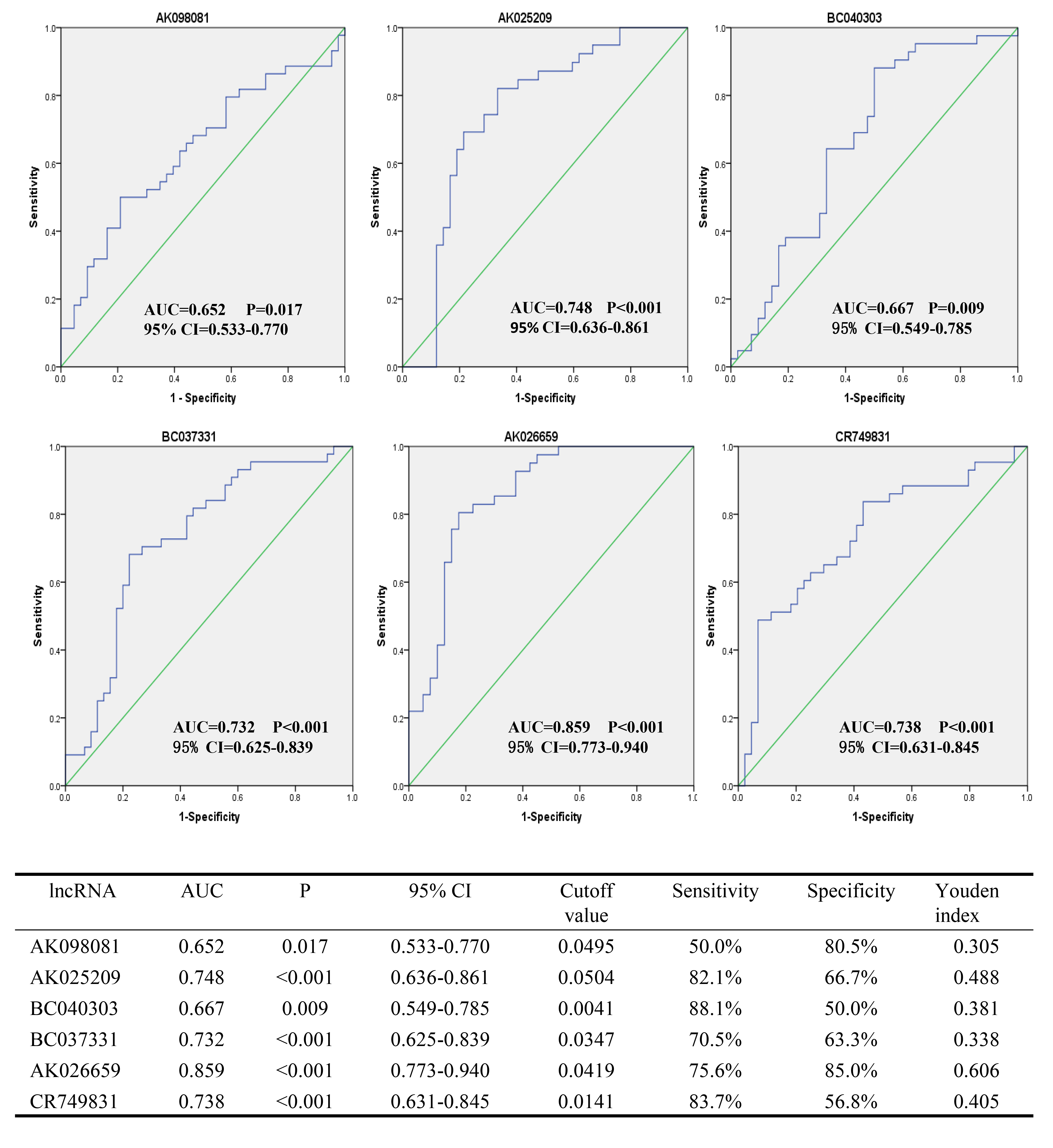

Supplement: S4 Fig — AUC: area under the ROC curve, CI: confidence interval. (TIF) [file pone.0164590.s004.tif]

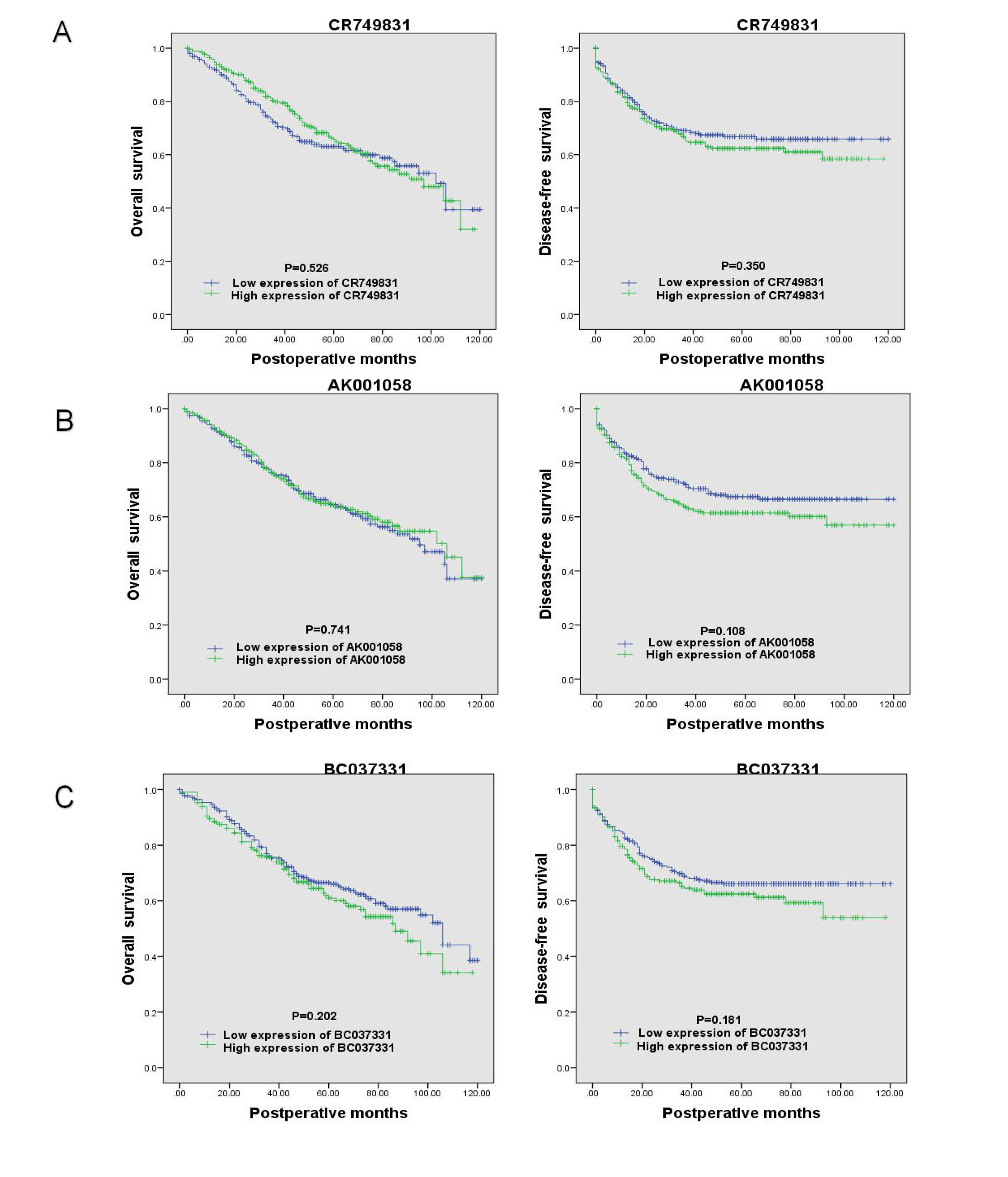

Supplement: S5 Fig — (A) Correlation of overall survival and disease-free survival with CR749831 expression. (B) Correlation of overall survival and disease-free survival of AK001058 expression. (C) Correlation of overall survival and disease-free survival of BC037331 expression. (TIF) [file pone.0164590.s005.tif]

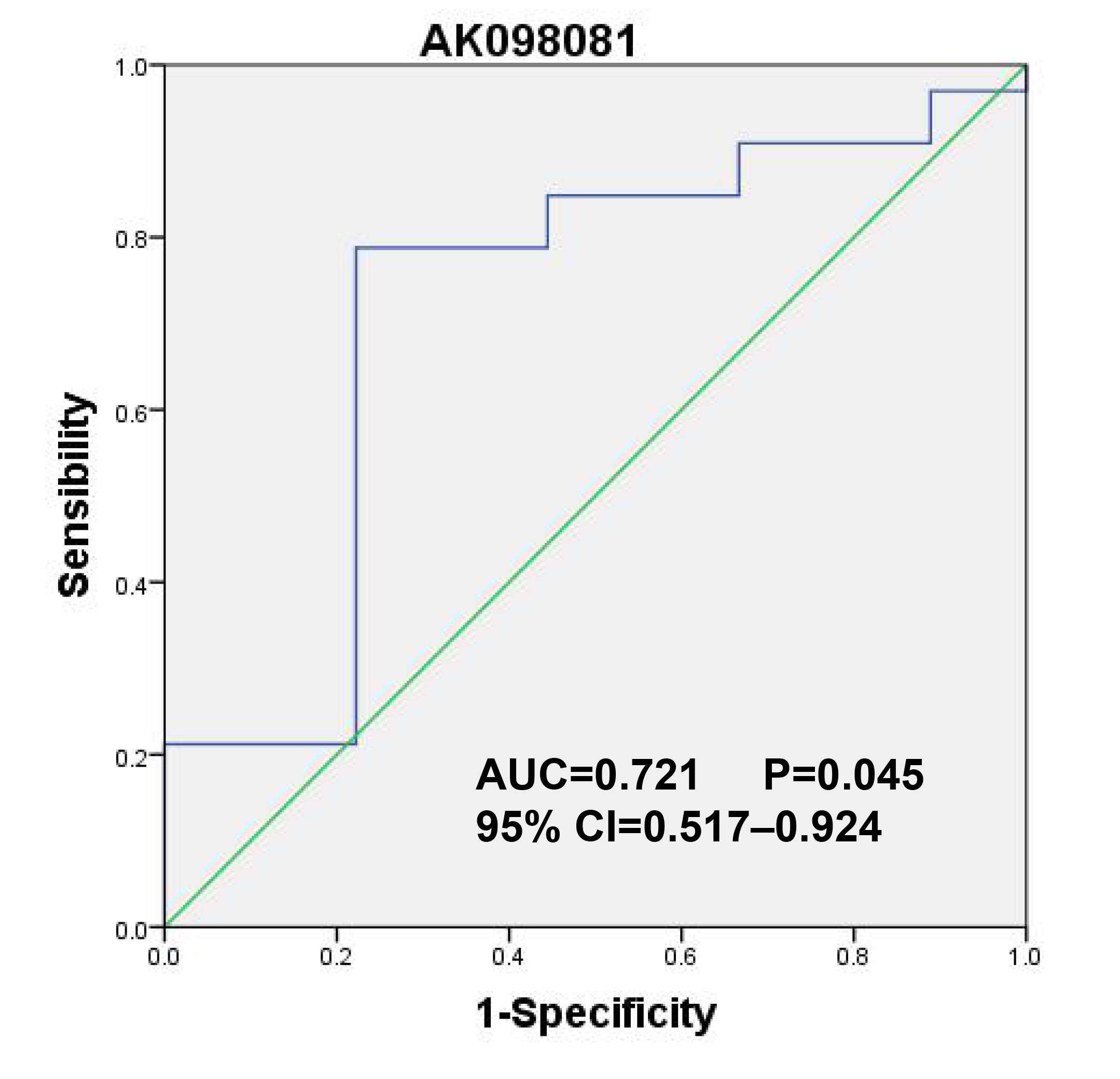

Supplement: S6 Fig — (TIF) [file pone.0164590.s006.tif]
